# Comparative Immunomodulatory Efficacy of Secukinumab and Honokiol in Experimental Asthma and Acute Lung Injury

**DOI:** 10.3390/ph18081108

**Published:** 2025-07-25

**Authors:** Andrei Gheorghe Vicovan, Diana Cezarina Petrescu, Lacramioara Ochiuz, Petru Cianga, Daniela Constantinescu, Elena Iftimi, Mariana Pavel-Tanasa, Codrina Mihaela Ancuta, Cezar-Cătălin Caratașu, Mihai Glod, Carmen Solcan, Cristina Mihaela Ghiciuc

**Affiliations:** 1 Department of Morpho-Functional Sciences II—Pharmacology and Clinical Pharmacology, Faculty of Medicine, Grigore T. Popa University of Medicine and Pharmacy of Iasi, 700115 Iasi, Romania; andrei-gheorghe.vicovan@umfiasi.ro (A.G.V.); diana.petrescu@umfiasi.ro (D.C.P.); cristina.ghiciuc@umfiasi.ro (C.M.G.); 2 Department of Pharmaceutical Technology, Faculty of Pharmacy, Grigore T. Popa University of Medicine and Pharmacy, 700115 Iasi, Romania; lacramioara.ochiuz@umfiasi.ro; 3 Department of Immunology, Faculty of Medicine, Grigore T. Popa University of Medicine and Pharmacy, 700115 Iasi, Romania; petru.cianga@umfiasi.ro (P.C.); d.constantinescu@umfiasi.ro (D.C.); elena_iftimi@umfiasi.ro (E.I.); mariana.pavel-tanasa@umfiasi.ro (M.P.-T.); 4 2nd Rheumatology Department, Clinical Rehabilitation Hospital, 700664 Iasi, Romania; codrina_ancuta@yahoo.com; 5 Rheumatology Department, Grigore T. Popa University of Medicine and Pharmacy, 700115 Iasi, Romania; 6 Advanced Research and Development Center for Experimental Medicine (CEMEX), Grigore T. Popa University of Medicine and Pharmacy of Iasi, 700115 Iasi, Romania; caratasu.catalin@umfiasi.ro; 7 Clinical Hospital CF Iasi, 700506 Iasi, Romania; mihai_glod@yahoo.com; 8 Department IX—Discipline of Histology, Embryology and Molecular Biology, Faculty of Veterinary Medicine, “Ion Ionescu de la Brad” University of Life Sciences, 700490 Iasi, Romania; 9 Pediatric Emergency Hospital Sf Maria, 700887 Iasi, Romania

**Keywords:** secukinumab, honokiol, asthma, acute lung injury, lipopolysaccharide, interleukin 17

## Abstract

**Background:** The study evaluates the immunomodulatory potential of secukinumab (SECU) and honokiol (HONK) in a murine model of allergic asthma complicated by acute lung injury (ALI), with an emphasis on modulating key inflammatory pathways. The rationale is driven by the necessity to attenuate Th17-mediated cytokine cascades, wherein IL-17 plays a critical role, as well as to explore the adjunctive anti-inflammatory effects of HONK on Th1 cytokine production, including IL-6, TNF-α, and Th2 cytokines. **Methods:** Mice were sensitized and challenged with ovalbumin (OVA) and lipopolysaccharide (LPS) was administrated to exacerbate pulmonary pathology, followed by administration of SECU, HONK (98% purity, C_18_H_18_O_2_), or their combination. Quantitative analyses incorporated OVA-specific IgE measurements, differential cell counts in bronchoalveolar lavage fluid (BALF), and extensive cytokine profiling in both BALF and lung tissue homogenates, utilizing precise immunoassays and histopathological scoring systems. **Results:** Both SECU and HONK, when used alone or in combination, display significant immunomodulatory effects in a murine model of allergic asthma concomitant with ALI. The combined therapy synergistically reduced pro-inflammatory mediators, notably Th1 cytokines, such as TNF-α and IL-6, as measured in both BALF and lung tissue homogenates. **Conclusions:** The combined therapy showed a synergistic attenuation of pro-inflammatory mediators, a reduction in goblet cell hyperplasia, and an overall improvement in lung histoarchitecture. While the data robustly support the merit of a combinatorial approach targeting multiple inflammatory mediators, the study acknowledges limitations in cytokine diffusion and the murine model’s translational fidelity, thereby underscoring the need for further research to optimize clinical protocols for severe respiratory inflammatory disorders.

## 1. Introduction

Acute lung injury (ALI) is a severe inflammatory condition defined by pulmonary inflammation, disruption of the alveolar–capillary barrier, and hypoxemia [1]. In individuals suffering from severe asthma, acute exacerbations may progress to ALI, resulting in considerable morbidity. Severe asthma and acute lung injury (ALI) share similarities, primarily characterized by acute respiratory failure and compromised oxidative status. The imbalance of redox systems favoring a dominant oxidative state in asthma is determinant for the pathophysiology of airflow limitation, hyperreactivity, and airway remodeling—key determinants of disease severity [2,3]—while antioxidant state is significantly diminished in patients with ALI [4,5]. A key factor for this impairment in both pathologies are the neutrophils [6,7] known to be mobilized and activated in high amounts trough Il-17 stimulation [8]. The inhibition of IL-17 has been demonstrated to ameliorate the lipopolysaccharide (LPS) exacerbated asthma in murine models [9,10,11,12] and, at the same time, to exert protective effects on LPS-induced ALI in mice [13]. Il-17 plays a key role in the cytokine storm associated with ALI of any etiology [14] modulating lung inflammation in LPS-induced ALI mice from a mechanistic point of view [15], with elevated Il-17 levels correlated with alveolar inflammation and a poor prognosis [16]. The pharmacological rationale for targeting IL-17 in certain asthma phenotypes is legitimated by its role in exacerbating severity characteristics; it reduces corticosteroids sensitivity [17] and induces hyperresponsiveness [18]. The beneficial effects of anti-Il-17 in LPS exacerbated asthma in murine models [9,10,11,12] and in the alleviation of ALI inflammation [19], possibly by reducing the expression of cytokines and oxidative stress [13], were already evaluated. Thus, targeting IL-17 has therapeutic potential in the treatment of acute respiratory failure associated either with severe asthma or with ALI.

In mice, the administration of IL-17 combined with a TH2 response results in increased neutrophilic inflammation within the pulmonary system. The data indicate that in patients with TH2-mediated asthma, extrinsic factors inducing IL-17A secretion may intensify neutrophilic inflammation and IL-8 secretion [20], consequently worsening lung pathology and progressing to ALI. Secukinumab (SEKU), an interleukin-17 (IL-17) inhibitor, has the potential to mitigate ALI by targeting key inflammatory pathways. By inhibiting IL-17, SEKU reduces the production of downstream pro-inflammatory cytokines, such as IL-6 and TNF-α, which are known to exacerbate lung injury. Moreover, SEKU decreases Th2 cytokines like IL-5, which are involved in eosinophilic inflammation and simultaneously has the ability to enhance the activity of anti-inflammatory cytokines, such as IL-10, promoting a shift toward resolution of inflammation and tissue repair [21]. IL-17 is associated with neutrophilic inflammation and steroid insensitivity in severe asthma, contributing to airway remodeling and hyperresponsiveness [22]. The combination of anti-IL-17A with corticosteroids has shown to significantly attenuate airway inflammation and hyperresponsiveness, suggesting a synergistic effect that could be more effective than either treatment alone in mixed-granulocytic asthma [23]. While both secukinumab and dexamethasone decrease IL-17 levels, dexamethasone also increases Th2 cytokines like IL-4, IL-5, and IL-13, which may not be desirable in all cases. Secukinumab specifically inhibits IL-5, which is beneficial in reducing eosinophilic inflammation without the broad immunosuppressive effects of corticosteroids [21].

The current approved anti-IL-5 and anti-IL-5R therapies are effective in reducing eosinophilic inflammation and asthma exacerbations. However, they do not target the IL-17 pathway, which is crucial in neutrophilic inflammation seen in severe asthma and ALI [24]. While the specificity of anti-IL-17 therapies may limit their effectiveness in conditions where multiple inflammatory pathways are involved, combining anti-IL-17 therapies with other anti-inflammatory treatments in respiratory diseases holds potential for synergistic effects.

**Figure 1:**
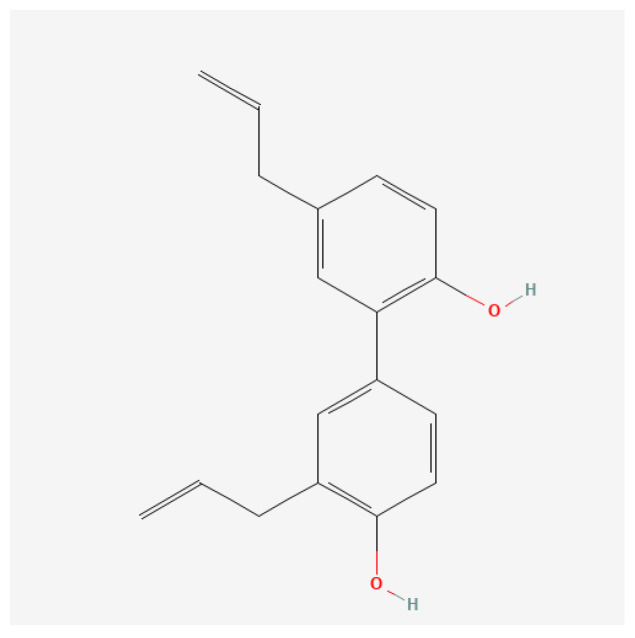
Honokiol chemical structure (source https://pubchem.ncbi.nlm.nih.gov/compound/72303, accessed on 21 July 2025).

Honokiol (HONK) is a biphenolic compound extracted from the bark and leaves of Magnolia species [25] with potent anti-inflammatory effects [26,27] (Figure 1). In asthma models, HONK reduces airway hyperresponsiveness and lung eosinophilia and decreases pro-inflammatory cytokines, such as TNF-α and IL-6, while increasing regulatory cytokines like IL-10 and TGF-β [28], and shows the potential to modulate the balance between Th17 and Treg cells, reducing the expression of inflammatory markers like TLR4 and NF-κB, which are elevated in asthma [29]. In ALI, HONK inhibits the activation of the NLRP3 inflammasome, which is crucial for the production of pro-inflammatory cytokines. This inhibition is partly mediated by the activation of the Nrf2 pathway, which reduces oxidative stress and inflammation [30].

Based on the existing data, we aimed to investigate the immunomodulatory effects of SECU and HONK in a comparative manner and in combination within mice exhibiting induced allergic asthma alongside concurrent ALI. Our study emphasizes the downstream implications for cytokine production, while evaluating the potential of SECU and HONK as therapeutic options for inflammatory lung conditions. The possibility of a synergistic therapeutic effect may facilitate the refinement of treatment protocols and enhance efficacy, while concurrently reducing the adverse effects commonly associated with currently approved therapies, such as corticosteroids.

## 2. Results

### 2.1. Effects of SECU, HONK and HONK + SECU Combined Treatment, Respectively, on Serum Levels of Ovalbumin (OVA)-Specific IgE

**Figure 2:**
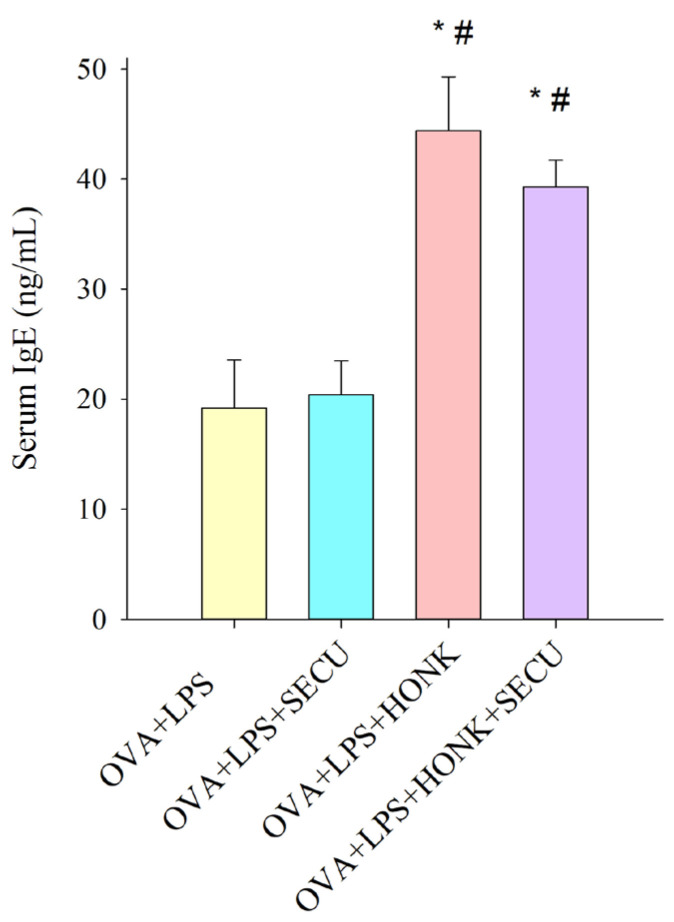
IgE concentration in serum (ng/mL). Values are expressed as mean ± standard error; *n* = 6. Significant differences (*p* < 0.05): * compared to OVA + LPS group; # compared to OVA + LPS + SECU group; OVA + LPS: positive disease control group; OVA + LPS + HONK: honokiol-treated group; OVA + LPS + SECU: secukinumab-treated group; OVA + LPS + HONK + SECU: combined honokiol- and secukinumab-treated group.

Figure 2 illustrates the concentration of serum OVA-specific IgE for each group, being statistically significant in OVA + LPS + HONK and in OVA + LPS + HONK + SECU groups compared to the SECU-treated group. This result confirms the presence of OVA sensitization in all groups and depicts the favorable role of HONK in the stimulation of IgE synthesis.

### 2.2. Effects of SECU, HONK, and HONK + SECU Combined Treatment, Respectively, on Bronchoalveolar Lavage Fluid (BALF) and on Lung Tissue Homogenate (LTH)

#### 2.2.1. Cell Count in BALF

**Figure 3:**
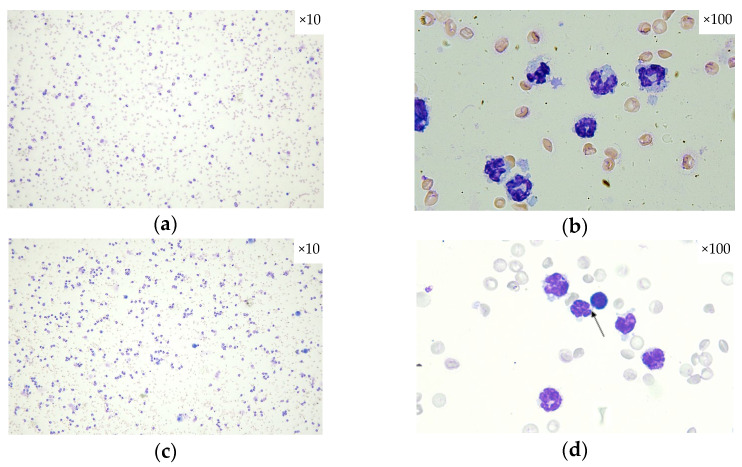

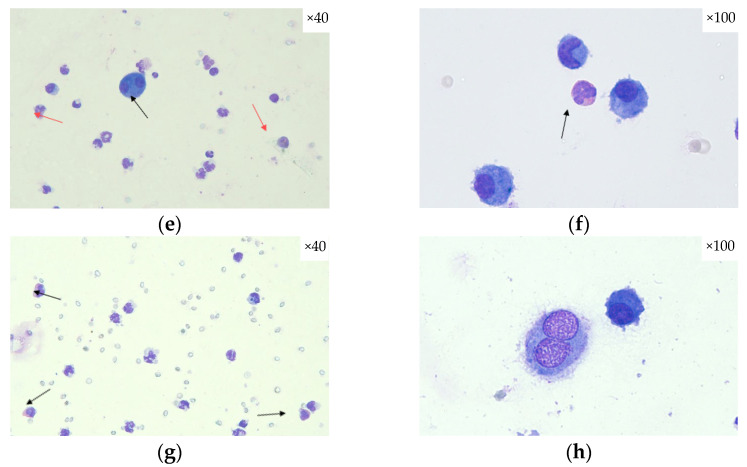
Differential cell count using optical microscopy: (**a**) OVA + LPS group: cellularity, ×10 dry objective; (**b**) OVA + LPS group: neutrophils, ×100 immersion objective; (**c**) OVA + LPS + SECU group: cellularity, ×10 dry objective; (**d**) OVA + LPS + SECU group: few neutrophils and one lymphocyte (arrow), ×100 immersion objective; (**e**) OVA + LPS + HONK group: binucleated alveolar macrophage (black arrow), neutrophils and eosinophils (red arrows), ×40 dry objective; (**f**) OVA + LPS + HONK group: alveolar macrophages and an eosinophil (arrow), ×100 immersion objective; (**g**) OVA + LPS + HONK + SECU group: neutrophils and eosinophils (arrows), ×40 dry objective; (**h**) OVA + LPS + HONK + SECU group: two alveolar macrophages, one binucleated, ×100 immersion objective.

In Figure 3, the modifications in inflammatory cell population types (macrophages, neutrophils, lymphocytes, and eosinophils) within BALF are depicted for each experimental group. An increased cellularity is present in all groups with the predominance of neutrophils and eosinophils.

**Figure 4:**
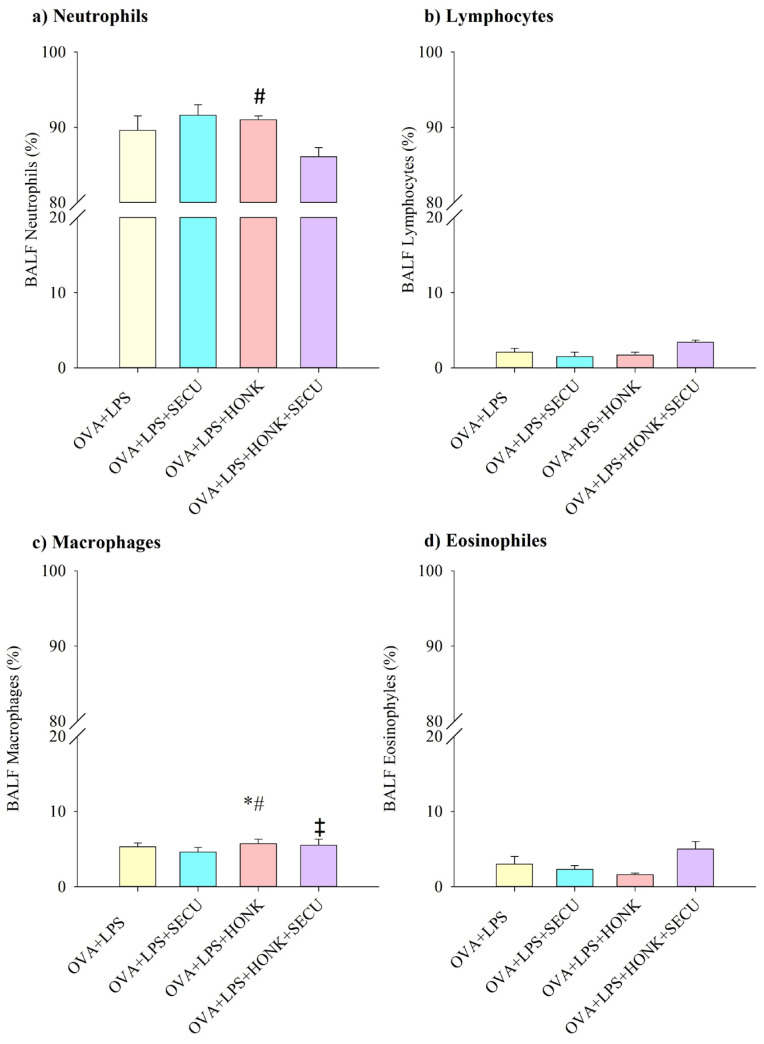
Differential cell counts in percentage for (**a**) macrophages, (**b**) neutrophils (**c**) lymphocytes, and (**d**) eosinophils. Values are expressed as mean ± standard error; *n* = 6. Significant differences (*p* < 0.05): * compared to OVA + LPS group; # compared to OVA + LPS + SECU group; ‡ compared to OVA + LPS + HONK group. OVA + LPS: positive disease control group; OVA + LPS + HONK: honokiol-treated group; OVA + LPS + SECU: secukinumab-treated group; OVA + LPS + HONK + SECU: combined honokiol- and secukinumab-treated group.

The assessment of the differential cell counts for neutrophils, lymphocytes, macrophages, and eosinophils indicated a notable percentage decrease in neutrophils within the OVA + LPS + HONK group in comparison to the OVA + LPS + SECU group (Figure 4a).

Alternatively, macrophages from OVA + LPS + HONK group were slightly but significantly increased compared to OVA + LPS and OVA + LPS + SECU groups. This effect of HONK is confirmed by the marginally decreased concentration of macrophages in OVA + LPS + HONK + SECU group in contrast to OVA + LPS + HONK group (Figure 4c).

No statistically relevant differences were observed in the percentage distributions of lymphocytes and eosinophils across the evaluated groups (Figure 4b,d).

#### 2.2.2. Th 1 Cytokines in BALF vs. In LTH

**Figure 5:**
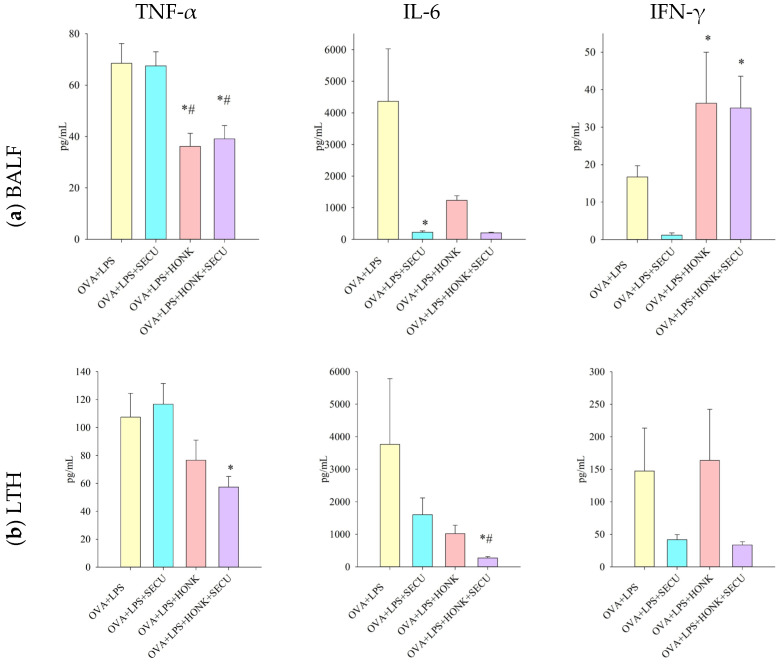
Th1 immune response-specific cytokines concentration (pg/mL) in (**a**) BALF and (**b**) LTH: TNF-α, IL-6, and IFN-γ. Values are expressed as mean ± standard error; *n* = 6. Significant differences (*p* < 0.05): * compared to OVA + LPS group; # compared to OVA + LPS + SECU group; OVA + LPS: positive disease control group; OVA + LPS + HONK: honokiol-treated group; OVA + LPS + SECU: secukinumab-treated group; OVA + LPS + HONK + SECU: combined honokiol- and secukinumab-treated group.

The impact of tested treatments and their association on Th1 immune response in BALF is miscellaneous. TNF-α level was significantly decreased by both HONK and HONK + SECU combination. However, it is difficult to estimate if SECU has any contribution to this effect, despite SECU’s doubtless potential to reduce IL-6 in monotherapy. Intriguingly, HONK administered solely or in association with SECU induced the increase in IFN-γ concentration in BALF (Figure 5a).

The evaluation of TNF-α concentration in LTH confirms the potential of the combined therapy to decrease its level. The ability of HONK + SECU to inhibit Th1 cytokines like IL-6 is even higher than that of SECU-only treatment, emphasizing the a potentially synergic effect for this association. Nevertheless, the poor statistical significance of the obtained IFN-γ concentration in LTH does not allow any comparison with its concentration in BALF for any of the analyzed groups (Figure 5b).

#### 2.2.3. Th 2 Cytokines in BALF vs. In LTH

**Figure 6:**
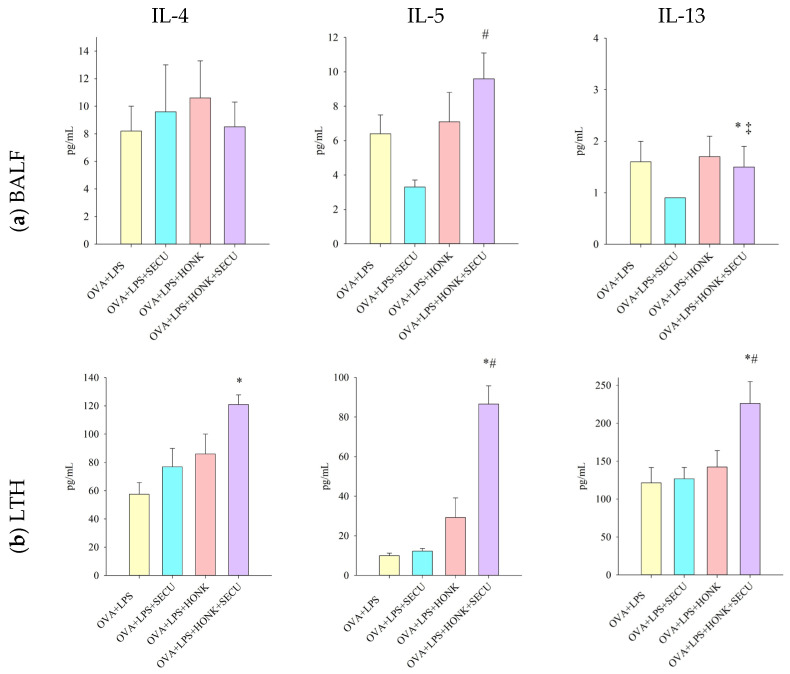
Th2 immune response-specific cytokine concentration (pg/mL) in (**a**) BALF and (**b**) LTH: IL-4, IL-5, and IL-13. Values are expressed as mean ± standard error; *n* = 6. Significant differences (*p* < 0.05): * compared to OVA + LPS group; # compared to OVA + LPS + SECU group; ‡ compared to OVA + LPS + HONK group. OVA + LPS: positive disease control group; OVA + LPS + HONK: honokiol-treated group; OVA + LPS + SECU: secukinumab-treated group; OVA + LPS + HONK + SECU: combined honokiol- and secukinumab-treated group.

Regarding Th2 immune response in BALF, the combined therapy induced an increase in Il-5 in contrast to the SECU-only treatment, though it is hard to assess the effect in comparation to positive disease control group. The combination HONK + SECU determined a modest decrease in Il-13, but this effect cannot be confirmed in comparison to HONK administration only. The influence exerted on Il-4 proved challenging to assess owing to the lack of statistical significance in the collected data (Figure 6a).

Both treatments appear to induce an increase in Il-4 concentration in LTH; however, the measured concentration is only statistically significant for the group that received combined treatment. As for IL-5 and IL-13, they were substantially increased by the association of SECU with HONK, partially confirming the results from BALF; moreover, this effect on IL-5 and IL-13 levels is much more evident than SECU administration only, suggesting a synergic outcome for the combined treatment (Figure 6b).

#### 2.2.4. IL-17 Cytokine in BALF vs. In LTH

**Figure 7:**
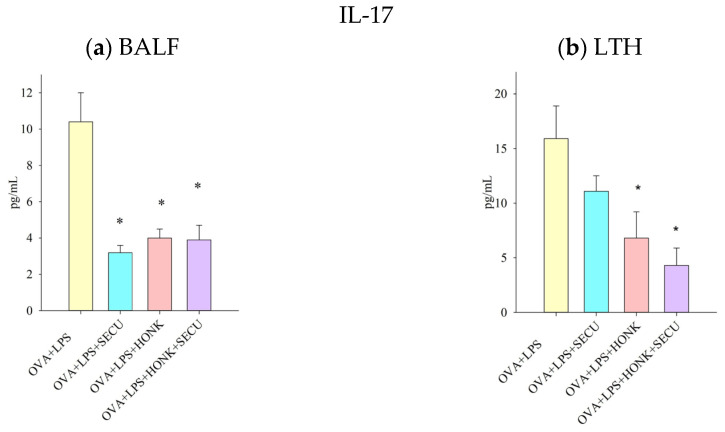
IL-17 cytokine concentration (pg/mL) in (**a**) BALF and (**b**) LTH. Values are expressed as mean ± standard error; *n* = 6. Significant differences (*p* < 0.05): * compared to OVA + LPS group; OVA + LPS: positive disease control group; OVA + LPS + HONK: honokiol-treated group; OVA + LPS + SECU: secukinumab-treated group; OVA + LPS + HONK + SECU: combined honokiol- and secukinumab-treated group.

The concentration of IL-17 in BALF and LTH, reflecting the participation of the Th17 immune response, has been diminished by both treatments applied either as standalone therapies or in combination. The variation in efficacy among treatments is, nevertheless, challenging to ascertain (Figure 7).

### 2.3. Histopathological Assessment of SECU, HONK, and HONK + SECU Combined Therapeutic Intervention

**Figure 8:**
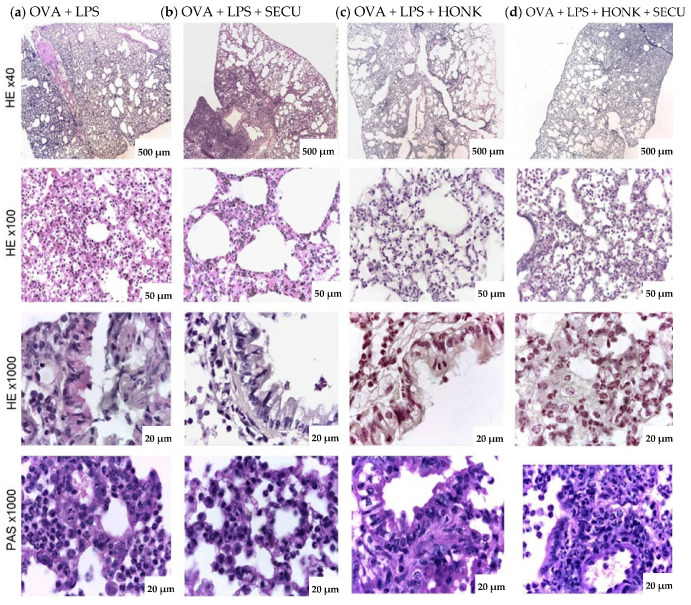
Representative histopathologic changes in H&E- (×40 or ×100) and PAS-stained (×1000) histological specimens sections of pulmonary and bronchial tissue from mice: (**a**) OVA + LPS: bronchioles with ulcerated epithelial cells, slightly congested blood vessels, thickened and infiltrated alveolar wall, excessive peri-bronchial inflammatory infiltrates with diffusely distributed eosinophils, neutrophils; (**b**) OVA + LPS + SECU: bronchioles lined by ciliated pseudo-stratified ciliated epithelium and moderate peri-bronchiolar and perivascular inflammatory infiltrates; (**c**) OVA + LPS + HONK: bronchioles lined by respiratory epithelium, alveolar walls and bullae of medium caliber, moderate to reduced inflammatory infiltrates; (**d**) OVA + LPS + HONK + SECU: lung tissue shows reduced lesions, moderate to reduced inflammatory infiltrates. OVA + LPS: positive disease control group; OVA + LPS + HONK: honokiol-treated group; OVA + LPS + SECU: secukinumab-treated group; OVA + LPS + HONK + SECU: combined honokiol- and secukinumab-treated group.

The lungs of mice in the OVA + LPS group exhibited increased cellular density within the pulmonary alveoli and in the peribronchiolar region, covering up to 60% of the parenchyma (see Figure 8). This dense area is considerably decreased in mice exposed to OVA + LPS + SECU, OVA + LPS + HONK, or OVA + LPS + HONK + SECU.

Histologic analysis of the lungs of mice in the OVA + LPS group reveals alterations in the goblet cell population of the pseudostratified ciliated epithelium that lines the respiratory tract, including an increase in both the number and the height of the cells. The epithelium is characterized by damaged intercellular junctions, observable by light microscopy, and perivascular and peribronchiolar edema. A notable finding in this particular group is the presence of polymorphonuclear, eosinophilic, lymphocytic, and peribronchial macrophages. Furthermore, the presence of polymorphonuclear cells has been observed in the lumen of the bronchi. Additionally, the septal wall and lumen of the pulmonary alveoli contain a variety of immune cells, including neutrophils, monocytes, eosinophils, alveolar macrophages, and lymphocytes. In addition to these findings, the pulmonary alveoli present a flattened appearance, and the septal wall has an increased diameter (Figure 8).

Histological analysis of the OVA + LPS + SECU group revealed a decrease in the number of neutrophils present in the proximity of the bronchi. The histologic characteristics of the pulmonary alveoli include a thinner wall, a reduced number of figurate elements in the lumen or septal wall, and a lower incidence of alveolar collapse. However, it was observed that a substantial area, constituting 15–20% of the lung, demonstrated characteristics comparable to those observed in the OVA + LPS group. In the OVA + LPS + HONK group, dense cellular infiltrates are maintained around the large bronchi and gradually decrease towards the bronchioles and pulmonary alveoli. In the OVA + LPS + HONK + SECU group, foci of cellular densification appear towards the periphery (occupying about 15–20%) of the pulmonary lobes, and in the rest of the sample, the infiltrates are reduced in number. As illustrated in Figure 8, the number of goblet cells in the experimental groups was reduced in the bronchi compared to the OVA + LPS group.

## 3. Discussion

In our study, HONK-treated mice determined the decrease in proinflammatory cytokines in response to OVA, including Th1-, and Th17-type cytokines, despite an increase in Th2-type cytokines. Other researchers found a similar profile of cytokines after the analysis of lung homogenates [28]. Thus, the modulation of B and T lymphocyte cytokine secretion induced by HONK occurs through a γ-aminobutyric acid type A-dependent mechanism, suggesting that symptoms and pathology of asthma may improve despite elevated Th2 cytokines [28]. In mice, LPS-rich OVA is known to evoke mixed Th1, Th2, and innate immune responses through the TLR-4 pathway, whereas LPS-free OVA evokes only a Th2 response [31].

SECU has the potential to modulate inflammatory cytokine release and decrease Th17 responses in a mouse model of ALI exacerbated by asthma [21].

To assess asthma severity and control, systemic markers like absolute neutrophil counts (ANC), blood eosinophil count (BEC), or C-reactive protein (CRP) can be correlated with bronchoalveolar lavage fluid (BALF) and lung tissue homogenate (LTH) analysis, which provides more rapid and precise insights into local airway inflammation. However, the lack of specificity for blood parameters like ANC, BEC, and CRP are not specific to asthma, can be influenced by various systemic conditions, and do not provide direct information about the airway environment, leading to potential misinterpretation. For instance, CRP levels are elevated in asthma patients but are also associated with other inflammatory conditions and do not correlate well with localized airway inflammation, making CRP levels a non-specific marker for asthma severity or control [32,33]. Blood eosinophil and neutrophil counts have shown poor specificity and sensitivity in predicting airway eosinophilic and neutrophilic inflammation, respectively. For instance, the optimal cut-off values for blood eosinophil count and fractional exhaled nitric oxide (FeNO) exhibited poor specificity (57% and 49%, respectively) in identifying airway inflammation phenotypes [34]. Blood parameters like ANC and CRP reflect systemic inflammation, which may not correlate with the specific inflammatory processes occurring in the lungs during ALI. This can lead to the misinterpretation of the severity and nature of lung injury [35]. Changes in blood parameters may not occur as rapidly as changes in lung pathology, potentially delaying the detection of acute changes in lung condition [36]. BALF analysis can identify the distinct molecular phenotypes of asthma, which are crucial for personalized treatment approaches. This includes the identification of protein–protein interactions and cytokine signaling pathways that differ between severe and non-severe asthma [37]. Moreover, LTH analysis is particularly useful for understanding the pathophysiology of asthma at a cellular level, allowing for the identification of specific inflammatory pathways, and potential therapeutic targets provide detailed information about the cellular composition and molecular markers of inflammation in the lung tissue [38]. Also, LTH can be particularly useful for understanding the specific inflammatory pathways active in ALI [39].

Cytokine trends in LTH generally align with those in BALF, although variations in absolute values and kinetics may exist, as BALF predominantly reflects cytokines released into the airway lumen, while lung tissue homogenate accounts for both extracellular and intracellular cytokines [40]. Despite its advantages, BALF faces challenges. The dilution of epithelial lining fluid during BAL can complicate the interpretation of acellular molecule measurements. Efforts to normalize these measurements using endogenous markers have shown mixed results, highlighting the need for careful consideration of dilution factors in BALF studies [41]. Additionally, while BALF provides a focused view of the alveolar space, it may not capture the full complexity of lung tissue responses.

LTH can capture cytokine levels from the entire lung tissue, providing a more complete picture of the inflammatory response. This is particularly useful in studies of infections or diseases that affect the lung parenchyma, such as *Pneumocystis carinii* infection, where significant cytokine activity was observed in lung homogenates but not in BALF [42].

BALF primarily samples the airway surface and may not capture the full spectrum of immune responses occurring deeper within the lung tissue. This limitation is evident in studies where BAL fluids failed to show significant increases in inflammatory cells or cytokines that were present in LTH [42].

LTH contains a mixture of cells and extracellular components from the entire lung tissue, which can dilute the specific signals of interest. This complexity can obscure the detection of localized immune responses and cytokine production that are more readily observed in BALF [41].

The rationale for simultaneously evaluating the same cytokines in both BALF and LTH was to facilitate a comprehensive understanding of the cytokine environment within the pulmonary system.

TNF-α contributes to the disruption of the bronchial epithelial barrier, which is crucial for maintaining lung homeostasis. This disruption is mediated through the loss of tight junction proteins, such as occludin and claudins, leading to increased permeability and further inflammation [43]. TNF-α also triggers alveolar epithelial dysfunction through p55 receptor-mediated death signaling, which is distinct from its proinflammatory signaling pathways. This mechanism leads to impaired alveolar fluid clearance and contributes to pulmonary edema, a critical feature of ALI [44].

In the context of ALI, inflammatory conditions promote the recruitment and differentiation of monocytes into the Gr-1+ subtype [45]. TNF-α-producing Gr-1+ monocytes significantly contribute to lung injury in sepsis [46], while TNF-α produced by alveolar macrophages play a critical role for the recruitment and activation of monocytes and neutrophils, essential for the advancement of LPS-induced ALI [47].

IL-6 contributes to epithelial cell dysfunction by promoting ferroptosis, a form of cell death characterized by lipid peroxidation and disrupted iron homeostasis. This process is mediated by reactive oxygen species (ROS) and is exacerbated by IL-6, leading to increased lung tissue damage [48]. IL-6 is produced by macrophages and dendritic cells, which are critical sources of pathogenic IL-6 in asthma [49].

However, our results show a decreased TNF-α (in both LTH and BLAF) and IL-6 (in LTH) concentration under HONK treatment (either in monotherapy or in association with SECU) in the context of slightly increased macrophages in OVA + LPS + HONK group.

The explanation behind these apparent contradictions in our results may be the increased IL-4 in LTH under the combined administration of SECU and HONK.

Macrophages exhibit distinct cytokine secretion patterns when polarized into M1 or M2 phenotypes. M1 macrophages are characterized by the production of pro-inflammatory cytokines including TNF-α and IL-6. These cytokines are crucial for initiating and sustaining inflammatory responses, which are essential for pathogen clearance and the initial stages of wound healing [50]. M2 macrophages stimulate the production of anti-inflammatory cytokines, such as IL-10 and transforming growth factor-beta (TGF-β), which help in resolving inflammation and promoting tissue repair [51].

IL-4 primarily signals macrophages through the IL-4 receptor, leading to M2 polarization trough activation of the STAT6 pathway, crucial for the transcription of M2-associated genes [52,53]. In a study involving engineered macrophages secreting IL-4, pulmonary transplantation of these macrophages in mice with ALI resulted in reduced lung inflammation, tissue injury, and mortality, suggesting even therapeutic potential for IL-4 in ALI through macrophage modulation [54].

IL-4 increase under combined treatment may exert potential benefits by enhancing neutrophil apoptosis in hypoxic conditions, which is a common feature in ALI. This effect is mediated through IL-4 receptor α (IL4Rα) signaling, which suppresses pro-inflammatory responses and aids in resolving lung injury [55].

In line with our study results, HONK inhibited the increased IL-6 and TNF-α in the serum and heart of CLP (cecal ligation and puncture)- and LPS-induced septic mice [56]. Treatment with HONK also decreased CSE (cigarette smoke extract)-induced inflammation by inhibiting the expression and secretion of TNF-α, IL-6, and IL-8 [57]. Intraperitoneally administrated, HONK significantly reduced the increases in serum TNF-α in a mouse model with associated ALI [58].

In the context of ALI, IL-5-driven eosinophilic inflammation can lead to increased vascular permeability and damage to the alveolar epithelium, contributing to the characteristic pulmonary inflammation and hypoxemia of ALI [59]. IL-5 enhances the recruitment of eosinophils to the lungs, where they contribute to airway inflammation and hyperresponsiveness, key features of asthma and ALI [60].

Although HONK-treated mice (previously sensitized to and challenged with OVA) showed a significant decrease in lung eosinophilia [28], in our study, the combined administration of SECU and HONK induced an obvious IL-5 increase. The key to understanding these apparent conflicting results may be the effects of IFN-γ increased by HONK administered solely or associated with SECU. IFN-γ was shown to reduce mucus production, chitinase activity, and eosinophilia (by inhibiting eosinophil generation in the bone marrow), indicating its ability to regulate systemic immune responses from the airway mucosal surface in mice [61]. IFN-γ also shifts the cytokine profile of invariant NKT (iNKT) cells from pro-asthmatic to protective IFN-γ production, thereby reducing airway hyperresponsiveness and eosinophilia [62].

IFN-γ is known to reduce the levels of IL-5, which are associated with airway inflammation and hyperreactivity in allergic asthma [63], but this effect is not reflected in our results, probably due to dynamic interplay among TH1 and Th2 immune responses.

Cytokines primarily secreted by the airway epithelium like IL-13 might be more readily detected and accurately quantified in BALF compared to LTH, where their signal could be diluted by the bulk of the tissue. This may explain why our results for IL-13 concentration are contradictory; HONK + SECU administration resulted in a statistically significant reduction in IL-13 within BALF, whereas the LTH assessment indicated a pronounced elevation of this cytokine.

Moreover, IL-13 has a dual nature; it is implicated in tissue repair processes following lung injury (it aids in matrix remodeling and the induction of epithelial-derived type 2 effector molecules, which are crucial for effective repair and recovery from lung damage) [64], and it has been shown to impair the integrity of the bronchial epithelial barrier by disrupting tight junctions, which can exacerbate asthma symptoms and potentially contribute to ALI. This disruption is mediated by type 2 innate lymphoid cells (ILC2s) and can lead to increased epithelial permeability, a hallmark of asthma pathogenesis [65].

Nevertheless, it is well-documented that IL-13 facilitates the synthesis of immunoglobulins, such as IgE [66]. Considering the elevated levels of IgE observed in our investigation, the administration of HONK + SECU seems to preferentially induce IL-13 production rather than inhibit it.

One possible reasoning for increased levels of IgE induced by HONK could be the increase in IL-13 and IL-4 (in lung tissue homogenate), which stimulates the maturation of B cells and significantly promotes IgE synthesis [67,68]. This Th2 cytokine increase usually is counterbalanced by the Th1 response in the context of ALI onset; however, in the current study, HONK suppressed Th1-specific cytokines like TNF-α. TNF-α influences the expression of IL-13 receptors, particularly IL-13Rα2, which act as a decoy receptor. This modulation can inhibit IL-13 bioactivity by preventing its signaling through IL-13Rα1, thereby reducing IL-13 release [69].

IL-17 is a key cytokine in the inflammatory response, contributing to neutrophil recruitment and activation, which are central to the pathogenesis of ALI and asthma exacerbations. Elevated levels of IL-17 have been associated with increased airway hyperresponsiveness and inflammation in severe asthma cases [70].

IL-17A is often cell-associated (produced by T cells in tissue); thus, Ma et al. found IL-17 significantly elevated in both BALF and lung in an OVA + LPS model, but absolute BALF IL-17 was modest (~tens of pg) in comparison to LTH. The BALF IL-17A may underestimate the total tissue cytokine content due to incomplete diffusion [71]. The evaluation of IL-17 in our research revealed almost the same pattern; for each group, the concentration of IL-17 was slightly increased in LTH in comparison to BALF, but HONK (alone or with SECU) was able to induce an evident decrease in IL-17 in both samples.

The inhibition of IL-17, through agents like anti-IL-17 antibodies, has demonstrated protective effects in experimental models of ALI, reducing inflammation, oxidative stress, and tissue remodeling [13,72]. However, in the context of microbial infections, which can exacerbate asthma, IL-17 may help in mounting an effective immune response, thereby preventing further lung damage [73]. Some studies even suggest that IL-17 could help in maintaining immune homeostasis and protecting against excessive type 2 inflammation, which is characteristic of eosinophilic asthma [70].

Finally, histopathological findings are in line with similar studies, indicating a significant reduction in pulmonary inflammation, goblet cell hyperplasia, and collagen accumulation following HONK treatment [28]. It is already known that imaging, such as micro-computer tomography (micro-CT) or ultra-high-resolution computed tomography (UHRCT), results significantly correlate with pathological findings in asthma [74,75] and have been shown to effectively detect and monitor changes in lung density and structure associated with LPS-induced ALI [76]. However, imaging investigations need expensive and dedicated equipment. Further research is needed to delineate the conditions under which IL-17 acts protectively versus pathologically, which could lead to more targeted and effective treatments for asthma and ALI.

### Study Limitations

One limitation of our study was that we focused only on the evaluation of specific cytokines, since the aim of our current study was to evaluate mainly the influence of HONK on the interplay of TH1, TH2, and TH17 immune responses; although, the evaluation of honokiol influence on Il-10 and TGF-β would enhance mechanistic insight.

We consider that another limitation is the measurement of cytokines only at the end of experiment. For an objective assessment, the influence of the administered therapeutic interventions on the interaction among various categories of immune responses should be ascertained dynamically across distinct phases of the progression of pathophysiological phenomena.

Another limitation is that the detection of cytokines in lung homogenates can be inconsistent, as shown in studies where cytokine levels in LTH did not always correlate with those in BALF. Certain cytokines may be present in lung homogenates but not in BALF, indicating that homogenates may not represent the lung cytokine environment accurately. Additionally, the presence of proteases and other enzymes in LTH can degrade cytokines, leading to the underestimation of their levels, thus impacting analytical accuracy [77]. LPS instillation causes a TNF-α spike in BALF within hours, reaching hundreds of pg/mL, while TNF in LTH may appear slightly lower when normalized by tissue mass [78].

Another limitation of the study is the lack of adjustment for multiple testing across cytokines. Future analyses could benefit from approaches such as the false discovery rate (FDR)/Bonferroni correction/another method correction or multivariate statistical models to better control for this issue.

Another limitation was the evaluation of local (pulmonary) immune response rather than the systemic inflammation through the assessment of blood parameters like ANC/BEC/CRP, since we focus on achieving a deeper understanding regarding the comparative immunomodulatory efficacy of secukinumab and honokiol.

## 4. Materials and Methods

### 4.1. Chemicals, Antibodies, and Reagents

The following reagents and pharmaceuticals from Sigma-Aldrich Chemie GmbH, Schnelldorf, Germany were utilized: aluminum hydroxide, lipopolysaccharide *Escherichia coli* O127:B8 (LPS), xylazine hydrochloride.

Honokiol (98% purity, C18H18O2; Mr = 266.3) was acquired from New Natural Biotechnology (Shanghai, China), Ovalbumin (GRADE V) was purchased from Sigma-Aldrich (St. Louis, MO, USA), phosphate-buffered saline solution from BioUltra, Protease Inhibitor Cocktail from Promega (Madison, WI, USA), NP-40 lysis buffer from Thermo Fisher Scientific (Lancashire, UK), and Ketamine Hydrochloride (commercial name Ketavet^®^ 100 mg/mL) from Zoetis (UK Limited, London, UK), saline solution (NaCl 0.9%) from Antibiotice SA (Iasi, Romania), carboxymethylcellulose sodium salt (CMC-Na high viscosity, 700–1500 mPa s; degree of substitution 0.60–0.95) from Fluka BioChemika (Buchs, Switzerland), polysorbate 80 (Tween^®^ 80, reagent grade lot: 19L0956375) from VWR Chemicals (Solon, OH, USA), xylazine hydrochloride from Sigma-Aldrich Chemie GmbH (Schnelldorf, Germany), Periodic Acid-Schiff (PAS) from Sigma-Aldrich Chemie GmbH (Schnelldorf, Germany), May-Grünwald 1.01424 from Sigma-Aldrich (Schnelldorf, Germany), and Giemsa (1.09204) from Sigma-Aldrich (Schnelldorf, Germany). The purified water was obtained with a distillation apparatus (GFL model 2004, serial number 11918315J, ProfiLab24 GmbH, Berlin, Germany).

Anti-IL-17 monoclonal antibody (Secukinumab, commercial name Cosentyx^®^) was acquired from Novartis Pharma GmbH (Nuremberg, Germany), the LEGEND MAX™ Mouse OVA Specific IgE ELISA Kit was acquired from BioLegend (San Diego, CA, USA), and the Luminex Mouse Discovery Assay 8-Plex encompassing IFN-γ, IL-4, IL-5, IL-6, IL-13, IL-17/IL17A, TNF-α, and VEGF was acquired from R&D Systems (Minneapolis, MN, USA).

### 4.2. Preparation of Honokiol Suspension and Ovalbumin Solution

The preparation of the HONK suspension pharmaceutical was realized by dispersing honokiol at a concentration of 0.5% (5 mg/g) within a 1% CMC-Na mucilage, formulated by diluting with distilled water and integrating 0.25% Tween^®^ 80, resulting in a suspension for oral administration.

The solution of ovalbumin (OVA) was obtained from OVA grade V. The intraperitoneal solution was obtained according to the protocol established by Debeuf et al. [79]; it was dissolved to achieve a final concentration of 20 μg/mL within 500 μL of sterile PBS per individual mouse, with the incorporation of aluminum hydroxide (alum) at a concentration of 2 mg/mL and agitated for 30 min at ambient temperature. The solution of ovalbumin (OVA) for aerosols was obtained as a 1% OVA solution diluted in PBS.

### 4.3. Experimental Design and Study Protocol

Criteria for animal selection: female mice were chosen for this experiment, since previous authors demonstrated a significantly heightened expression of allergic airway inflammation post-OVA challenge in female mice in contrast to their male counterparts [80].

The protocol of the study was approved by the Institutional Ethics Committee of “Grigore T. Popa” University of Medicine and Pharmacy in Iaşi, Romania (approval no. 332/17.09.2023). The study was in compliance with Research Law No. 206/2004 (published by the National Council for Ethics in Scientific Research, Technological Development, and Innovation) and in compliance with the ARRIVE guidelines.

The effects of OVA-only [20,81] or LPS-only [82,83] exposure on immune response were already evaluated previously. The study design is aimed to bring more clarity in a particular murine model of LPS-induced ALI overlapped on OVA-induced allergic pulmonary inflammation, as this type of experimental ALI showed an increased level of severity [84].

Thirty adult female BALB/c mice (weighing from 17 to 23 g) were purchased from the “Cantacuzino” Institute in Bucharest. The animals (randomized in groups with *n* = 6 mice per group) were housed in sterile cages, with sufficient food and water, at relative constant temperature (22 °C ± 3 °C), humidity (55% ± 5%), and a circadian rhythm of 12 h of light and 12 h of darkness. Mice were acclimatized for seven days prior to the initiation of experimental study.

The experiment was carried out at the CEMEX facility at our university. The experimental groups were:

- Group 1 (OVA-LPS) (positive disease control): received inhaled ovalbumin and instilled LPS;
- Group 2 (OVA-LPS + SECU): received inhaled ovalbumin and instilled LPS and subcutaneous administration of secukinumab;
- Group 3 (OVA-LPS + HONK): received inhaled ovalbumin and instilled LPS and oral gavage treatment with honokiol;
- Group 4 (OVA-LPS + HONK + SECU): received inhaled ovalbumin and instilled LPS and both oral gavage treatment with honokiol and subcutaneous administration of secukinumab.

In line with the 3Rs principles (Replacement, Reduction, Refinement) and institutional guidelines, we minimized the number of animals used, while still meeting power requirements. All cytokines were assayed from the same animals, avoiding additional cohorts. For LPS-induced ALI in the Balb/c mice model, previous experimental studies of other authors on BALF cytology and cytokine response (IL-1β, IL-17 and TNF-α) evaluated individual exposure groups to LPS with or without anti-Il-17 [13], using 6 animals/group [9,85].

**Figure 9:**
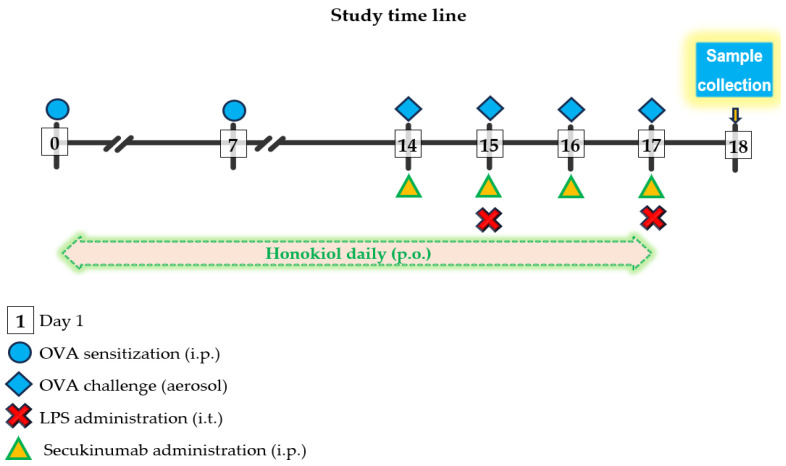
The chronological framework of the procedures.

The mice were sensitized and challenged with OVA i.p. injection and aerosolized, respectively, in accordance with the methodology described by Debeuf et al. [79]. The mice were sensitized on days 0 and 7 with 0.2 mL per mouse i.p. injections of the OVA/alum mixture. The mice were challenged on days 14, 15, 16, and 17, with 10 mL of a 1% OVA solution diluted in PBS in aerosols for 25 min in the inhalation chamber (Figure 9).

Acute lung injury was induced with LPS on days 15 and 18, under general anesthesia with i.p. ketamine (80 mg/kg) and xylazine (10 mg/kg) injection, in accordance with the methodology described by Ehrentraut et al. [86]. The administration of intratracheal LPS was conducted one hour following the OVA aerosol exposure.

The dose of 10 mg/kg SC SECU showed benefits [87] and the ability to induce immunomodulatory effects in respiratory disease when used in Balb/c mice [21]. In an OVA-induced mouse model of allergic asthma 50 mg/kg PO of HONK reduced the airway inflammation and suppressed the production of inflammatory cytokines [88].

SECU (10 mg/kg) was administered subcutaneously from day 14 to 17, one hour before the administration of each OVA aerosol. The dose of SECU was established based on similar studies conducted on rodents [87,89].

HONK (50 mg/kg) was administrated by oral gavage, daily, 1 h before OVA challenge for OVA + LPS + HONK group and 1 h before SECU administration for OVA + LPS + HONK + SECU groups, from the first day of OVA exposure (day 0) and lasting until day 18.

On the 18th day, all mice were euthanized under profound anesthesia (resulting from an overdose of ketamine and xylazine).

### 4.4. Assessment of Serum OVA-Specific IgE Levels

Whole blood samples (of 0.5 mL) were centrifuged at 3000 rotations/minute, for 10 min at 4 °C, to isolate serum, which was further used for the determination of OVA-specific IgE utilizing a Legend Max Mouse OVA Specific IgE ELISA kit (Biolegend, San Diego, CA, USA). Absorbance was measured using a microplate reader (Infinite 200 PRO M Plex Tecan plate reader, Tecan, Grödig, Austria).

### 4.5. Assessment of Cell Count and Cytokines in Bronchoalveolar Lavage Fluid (BALF)

A total volume of 1.5 mL of bronchoalveolar lavage fluid (BALF) was collected on ice-cold phosphate-buffered saline (PBS) by three sequential infusions and aspirations of 0.5 mL each through tracheal cannula. The cell suspensions from each mouse were centrifuged at 250× *g*, for 5 min at 4 °C, to separate the cells from fluids, and aliquots of 5 µL from each residual cell pellet were used for differential cell counting evaluation, after subsequent May–Grunwald–Giemsa staining. Using an optical microscope, the mean cell count was determined by examining 10 fields with a dry objective lens (400×), while cell identification was based on the analysis of 200 cells with an oil-immersion objective lens (1000×). Cell differential assessments were performed blinded by two independent personnel. The supernatant was further centrifuged at 10,000× *g*, for 15 min at 4 °C, and aliquots of 150 µL were frozen at −80 °C for cytokine analysis. The cytokine panel of RD-LXSAMSM-08 Luminex Mouse Discovery Assay 8-Plex included interferon (IFN)-γ, interleukin (IL)-4, IL-5, IL-6, IL-13, IL-17/IL17A, tumor necrosis factor (TNF)-α, vascular endothelial growth factor (VEGF). The results are reported in pg/mL.

### 4.6. Assessment of Cytokines in Lung Homogenates

Post-sacrifice, the right lung of each mouse was weighed and immersed for 15 min in 200 µL of NP-40 lysis buffer mixed with a Protease Inhibitor Cocktail and kept on ice. The tissues were manually homogenized on ice with a 1 mL Wheaton Tenbroeck tissue grinder. Lung homogenates were further centrifuged at 10,000× *g* for 15 min at 4 °C, and aliquots of 60 µL were frozen at −80 °C for cytokine analysis. The cytokine panel of LXSAMSM-08 Luminex Mouse Premixed Multi-Analyte kit included IFN-γ, IL-4, IL-5, IL-6, IL-13, IL-17/IL17A, TNF-α, VEGF. The samples underwent a dilution of 1.25 times in RD 6-52 and were processed according to the specifications provided by the manufacturer of the kit LXSAMSM-08 Luminex Mouse Premixed Multi-Analyte kit (R&D Systems, Minneapolis, MN, USA) in combination with a Gen-Probe Luminex 100/200 xMAP platform (Austin, TX, USA).

### 4.7. Histological Analysis

The left lung’s lower lobe from each mouse was weighed before being immersed in 10% buffered formalin for a duration of 24 h, followed by dehydration through sequential exposure to 70–95% ethanol and 100% xylene, ultimately being paraffin-embedded. Lung sections of 4 µm were stained with hematoxylin and eosin (H&E) for evaluating infiltrating immune cells and epithelial thickening and with Periodic Acid-Schiff (PAS) to assess airway epithelial goblet cell abundance using a previously reported scoring system [90]. Extent and severity of lung inflammation was evaluated using a semi-quantitative scoring system on a scale from 0 to 4, as follows: 0, no cells; 1, few cells; 2, a ring of cells (one cell layer deep); 3, a ring of cells (two to four cells deep); and 4, a ring of cells (more than four cells deep). Mucus secretion was evaluated using a scale from 0 to 4, as follows: 0 for <0.5% PAS-positive cells; 1 for <25%; 2 for 25–50%; 3 for 50–75%; and 4 for >75%. A Leica microscope was used to analyze four different sections of each mouse to evaluate the histological architecture and to score the presence of infiltrating immune cells and goblet cells. 

### 4.8. Statistical Analysis

All experimental data are expressed as means ± standard error (SE), with bar graphs depicting SE bars for clarity. Statistical significance among groups was assessed using the parametric one-way analysis of variance (ANOVA), followed by either the Holm–Sidak procedure for multiple comparisons or the non-parametric Kruskal–Wallis one-way analysis of variance on ranks. We used one-way ANOVA followed by the Holm–Sidak procedure (or Kruskal–Wallis with appropriate post hoc tests) to adjust for multiple comparisons within each cytokine—that is, across experimental groups for a given cytokine. However, we did not apply a correction for multiplicity across the multiple cytokine comparisons (i.e., testing several cytokines independently), which may increase the risk of Type I error due to high differences among the ranges of normal values of each cytokine. The analysis was conducted using the Statistical Package for the Social Sciences (SPSS) version 27.0 (SPSS Inc., Chicago, IL, USA) software. A *p*-value of less than 0.05 was considered statistically significant.

## 5. Conclusions

The research establishes that both SECU and HONK, when used alone or in combination, display significant immunomodulatory effects in a murine model of allergic asthma concomitant with ALI. The combined therapy appears to synergistically reduce pro-inflammatory mediators, notably Th1 cytokines, such as TNF-α and IL-6, as measured in both BALF and lung tissue homogenates, while also modifying Th2 cytokine profiles with subtle increases in IL-4 and more pronounced elevations of IL-5 and IL-13. Histopathological assessments revealed an attenuation of lung inflammation, reduced goblet cell hyperplasia, and diminished cellular infiltrates particularly around bronchi and pulmonary alveoli, which further substantiates the beneficial impact of the treatments on lung tissue structure and function.

Moreover, the study elucidates that while the specificity of anti-IL-17 therapies, such as SECU is advantageous, their strategic combination with the anti-inflammatory compound HONK can enhance overall therapeutic efficacy by targeting multiple inflammatory pathways concurrently. The cytokine profiling, bolstered by the dual evaluation in BALF and LTH, provides critical insights into the complex immunological landscape underlying ALI and asthma, albeit with recognized limitations regarding murine model applicability and cytokine detection consistency. Overall, these findings suggest that a combinatorial approach holds promise for refining therapeutic protocols to alleviate airway inflammation in severe respiratory disorders.

## Data Availability

All the data from this study are published in the paper.
